# Advanced Remote Sensing and AI Techniques in Agriculture and Forestry

**DOI:** 10.3390/plants15172706

**Published:** 2026-09-03

**Authors:** Rui-Feng Wang, Kangning Cui

**Affiliations:** 1 Department of Crop and Soil Sciences, College of Agriculture and Environmental Sciences, University of Georgia, Tifton, GA 31793, USA; 2 Department of Computer Science, Wake Forest University, 1834 Wake Forest Road, Winston-Salem, NC 27109, USA; 3 Department of Data Science, City University of Hong Kong (Dongguan), 8 Gaoxiong Road, Songshan Lake High-Tech Industrial Development Zone, Dongguan 523808, China

## 1. Introduction

Rapid advances in sensing, computation, and automation are reshaping the ways in which plants and agricultural systems are observed, analyzed, and managed [1,2,3,4,5,6,7,8]. Conventional plant measurements and field surveys remain indispensable for acquiring reliable biological and agronomic information, but they are often labor-intensive, time-consuming, and difficult to scale across large populations, heterogeneous environments, and repeated observations [9,10]. Meanwhile, agricultural production is increasingly required to address growing demands for food production while coping with resource constraints, environmental variability, and climate-related risks [11,12,13]. These challenges have created a strong need for efficient approaches capable of acquiring plant information at multiple spatial and temporal scales and converting these observations into quantitative and actionable knowledge.

Remote sensing and artificial intelligence (AI) have consequently become increasingly important tools in modern plant science and precision agriculture [14,15,16]. Satellite, unmanned aerial vehicle (UAV), proximal, hyperspectral, multispectral, and microscopic imaging systems provide complementary information ranging from regional vegetation patterns to individual plants, organs, tissues, and cells [9,17,18,19,20,21]. In parallel, machine learning and deep learning methods enable increasingly sophisticated extraction of structural, physiological, and phenotypic information from these data [22,23,24,25,26]. AI is also being integrated with robotics, sensor fusion, navigation, and data-driven modeling, extending its role beyond image interpretation toward autonomous sensing, prediction, and agricultural operations [27,28,29,30,31,32,33,34,35,36]. The resulting convergence of sensing technologies and intelligent computation provides new opportunities for crop monitoring, plant phenotyping, stress assessment, forest characterization, quality prediction, and automated agricultural management.

Nevertheless, the practical application of these technologies continues to face important challenges. Agricultural and natural environments are highly heterogeneous, and changes in illumination, canopy structure, growth stage, sensor configuration, environmental conditions, and spatial scale can substantially affect data distributions and model performance [37,38,39,40,41]. The availability of accurately labeled datasets remains limited for many plant applications, while models developed under specific experimental conditions may not transfer reliably to new crops, locations, seasons, or sensing platforms [42]. In addition, the transition from accurate experimental models to deployable systems requires consideration of computational efficiency, multi-source information integration, navigation reliability, edge implementation, and the biological relevance of predicted traits [43].

Against this background, this Special Issue brings together ten contributions that illustrate recent progress in remote sensing, artificial intelligence, computer vision, robotic platforms, plant phenotyping, physiological trait estimation, and data-driven agricultural applications. The contributions span a particularly broad range of observational scales, from regional forest biomass mapping and UAV-based tree monitoring to greenhouse fruit assessment and microscopic plant-cell segmentation. Overall, these studies demonstrate how advances in sensing and AI are progressively connecting plant observation with quantitative characterization, prediction, and intelligent decision-making.

## 2. Overview of the Special Issue

This Special Issue presents recent advances in the integration of artificial intelligence, remote sensing, robotics, and plant phenotyping across multiple agricultural and ecological scales. Xu et al. [44] provide a broad review of AI applications in crop production, covering biotic stress monitoring, soil management, precision operations, supply-chain optimization, and climate-resilient agriculture. Their synthesis highlights model generalization, data bias, implementation barriers, and sustainability as key constraints on broader deployment. This system-level perspective provides an appropriate context for the more application-oriented studies included in the Special Issue.

Autonomous sensing platforms are addressed from both aerial and ground perspectives. Dong et al. [45] review agricultural UAV navigation across sensing, localization, mapping, planning, and control, emphasizing the transition of UAVs from passive imaging platforms toward task-oriented systems for monitoring, spraying, and other field operations. Complementarily, Zhang et al. [46] examine ground mobile robots for high-throughput plant phenotyping through a perception–decision–action framework, with particular attention to multimodal sensing, navigation, phenotypic analysis, robotic intervention, and edge deployment. Together, these studies show that agricultural platforms are increasingly evolving toward integrated systems in which sensing and autonomous operation are tightly coupled.

Several contributions focus on robust retrieval of plant physiological traits from spectral information. Jin et al. [47] develop a hyperspectral framework for estimating layer-specific leaf nitrogen content in potato canopies using fractional-order derivatives and optimized spectral indices, demonstrating improved sensitivity to vertical canopy heterogeneity. Chen et al. [48] address chlorophyll inversion under heterogeneous shading conditions by formulating illumination variation as a conditional domain-shift problem and introducing a domain-adaptation framework. Both studies move beyond fixed-condition spectral modeling by explicitly addressing structural or environmental sources of variability that limit model transferability.

Multi-source remote sensing is further explored in forest- and tree-level applications. Qian et al. [49] combine Sentinel-2, ALOS-2 PALSAR-2, topographic information, deep learning, and geostatistical residual correction for regional forest aboveground biomass mapping. At a finer scale, Guo et al. [50] integrate UAV-based RGB and multispectral imagery through cross-modal attention for wild ginkgo crown segmentation. These studies demonstrate the value of combining complementary sensing sources to improve vegetation characterization from regional forest structure to individual-tree delineation.

Computer vision applications in this Special Issue extend from robotic crop perception to microscopic plant analysis. Li et al. [51] propose a unified instance-segmentation framework for maturity grading of tomatoes and sweet peppers, addressing the limitations of crop-specific models while maintaining real-time suitability for robotic deployment. Zhou et al. [52] develop a multi-scale attention-based U-Net for moso bamboo cell segmentation, targeting weak boundaries, dense structures, and strong morphological variation in microscopic imagery. Together, these contributions illustrate the scalability of AI-based image analysis across markedly different biological levels.

Finally, Zeng et al. [53] extend data-driven plant analysis beyond imaging by integrating physiological, canopy, and metabolic variables with feature screening and a back-propagation neural network for apple-quality prediction. This work demonstrates how AI can connect plant functional traits with economically relevant quality outcomes and support more targeted orchard management.

Collectively, the ten contributions highlight three converging directions: the integration of heterogeneous sensing sources, the increasing emphasis on robustness and transferability, and the evolution from isolated perception tasks toward deployable intelligent systems. These trends reflect a broader shift in plant science from data acquisition alone toward quantitative interpretation and operational decision support.

## 3. Conclusions and Perspectives

The contributions to this Special Issue demonstrate a clear shift in plant science from isolated sensing and prediction tasks toward integrated, data-driven systems. Across remote sensing, computer vision, robotics, and physiological modeling, a common trend is the increasing use of multimodal and multi-source information to improve plant characterization across spatial scales.

At the same time, robustness and transferability remain major constraints. Variations in crop type, illumination, canopy structure, environment, season, and sensing configuration can substantially affect model performance. Future work should therefore place greater emphasis on cross-environment validation, efficient learning from limited annotations, standardized evaluation, and biologically interpretable modeling rather than accuracy under narrowly defined experimental conditions.

A further priority is the closer integration of sensing, AI, and autonomous platforms. Progress in UAVs, ground robots, and robotic vision indicates that agricultural intelligence is moving toward closed-loop systems that connect perception with decision-making and action. Advancing this transition will require coordinated development of algorithms, sensing hardware, navigation, edge computing, and task-specific agronomic knowledge.

Overall, the studies collected here show that remote sensing and AI are becoming increasingly central to quantitative plant phenotyping, crop and forest monitoring, and intelligent agricultural management. Continued progress will depend on developing methods that are not only accurate, but also transferable, efficient, and deployable under realistic biological and field conditions.

## Data Availability

No new data were created or analyzed in this study. Data sharing is not applicable to this article.
